# Recombinant production and characterization of full-length and truncated β-1,3-glucanase PglA from *Paenibacillus* sp. S09

**DOI:** 10.1186/1472-6750-13-105

**Published:** 2013-11-28

**Authors:** Rui Cheng, Jinping Chen, Xiaohong Yu, Yang Wang, Shiming Wang, Jianfa Zhang

**Affiliations:** 1 Center for Molecular Metabolism, Nanjing University of Science & Technology, 200 Xiaolingwei, Nanjing 210094, China

**Keywords:** β-1,3-glucanase, *Paenibacillus*, Carbohydrate-binding, Ig-like domain, Thermostability

## Abstract

**Background:**

β-1,3-Glucanases catalyze the hydrolysis of glucan polymers containing β-1,3-linkages. These enzymes are of great biotechnological, agricultural and industrial interest. The applications of β-1,3-glucanases is well established in fungal disease biocontrol, yeast extract production and wine extract clarification. Thus, the identification and characterization of novel β-1,3-glucanases with high catalytic efficiency and stability is of particular interest.

**Results:**

A β-1,3-glucanase gene designated *PglA* was cloned from a newly isolated strain *Paenibacillus* sp. S09. The gene *PglA* contained a 2631-bp open reading frame encoding a polypeptide of 876 amino acids which shows 76% identity with the β-1,3-glucanase (BglH) from *Bacillus circulans* IAM1165. The encoded protein PglA is composed of a signal peptide, an N-terminal leader region, a glycoside hydrolase family 16 (GH16) catalytic domain and a C-terminal immunoglobulin like (Ig-like) domain. The *Escherichia coli* expression system of PglA and five truncated derivatives containing one or two modules was constructed to investigate the role of catalytic and non-catalytic modules. The pH for optimal activity of the enzymes was slightly affected (pH 5.5-6.5) by the presence of different modules. However, the temperature for optimal activity was strongly influenced by the C-terminal domain and ranged from 50 to 60°C. Deletion of C-terminal domain resulted in obviously enhancing enzymatic thermostability. Specific activity assay indicated that PglA specifically hydrolyzes β-1,3-glucan. Insoluble β-1,3-glucan binding and hydrolysis were boosted by the presence of N-and C-terminal domains. Kinetic analysis showed that the presence of N-and C-terminus enhances the substrate affinity and catalytic efficiency of the catalytic domain toward laminarin. Carbohydrate-binding assay directly confirmed the binding capabilities of the N-and C-terminal domains.

**Conclusions:**

This study provides new insight into the impacts of non-catalytic modules on enzymatic properties of β-1,3-glucanase. Activity comparison of full-length PglA and truncated forms revealed the negative effect of C-terminal region on thermal stability of the enzyme. Both the N-and C-terminal domains exerted strong binding activity toward insoluble β-1,3-glucan, and could be classified into CBM families.

## Background

β-1,3-Glucanase catalyzes the hydrolysis of β-1,3-glucosidic bonds existing in β-1,3-glucan, which is the main cell wall component of yeast and filamentous fungi, and a major structural and storage polysaccharides (laminarin) in marine macroalgae, and produced as exopolysaccharide by some bacteria [1]. β-1,3-Glucanases are widely distributed among from plants, marine animals, fungi and bacteria. According to the divergence of amino acid sequence, β-1,3-glucanases from plants are divided into glycosyl hydrolase (GH) family 17 while the bacterial enzymes are grouped into GH16 family [2]. β-1,3-Glucanases synthesized in plants are highlighted because of the plant defense against fungal pathogens. Additionally, they also play roles in the normal biological and developmental processes in plants and fungi. In bacteria, β-1,3-glucanases participate in the degradation of polysaccharides which are utilized as an nutrient source [3,4].

There has been a great deal of interest in research of bacterial β-1,3-glucanases. They have shown potential biotechnical applications in structural analysis of yeast and fungal cell wall [5], and preparation of protoplasts [6]. β-1,3-Glucanase could also act as a potential biocontrol agent against pathogenic fungi in agriculture [7], and a useful tool to prevent slime production and undesirable yeast growth during vinification in the wine-making process [8]. In relation to pharmaceutical application, the soluble β-1,3-glucan oligosaccharides digested by β-1,3-glucanase are potential immuneactivators [9]. Based on the substrates hydrolyzing mechanisms, β-1,3-glucanases are classified into two types: exo-1,3-β-D-glucanases (EC 3.2.1.58) which release glucose residues from the non-reducing end of a substrate, and endo-1,3-β-D-glucanases (EC 3.2.1.6 and EC 3.2.1.39) which catalyze the hydrolysis of internal β-1,3-glucosidic bonds at random sites and release short oligosaccharides [10]. There are many β-1,3-glucanases that have been purified and characterized from different bacterial species, including *Bacillus*[11], *Paenibacillus*[12,13], *Micromonospora*[6], *Streptomyces*[7] and *Cellulosimicrobium*[14]. Proposed physiological functions of bacterial β-1,3-glucanases vary depending on the source and structure of enzyme [15]. In view of the industrial and biotechnological importance of β-1,3-glucanase, there is of great potential interest in identifying and characterizing novel β-1,3-glucanases.

In the present study, a novel β-1,3-glucanase gene was identified by from *Paenibacillus* sp. S09, which was isolated from the rhizosphere of oat plants. The deduced amino acid sequence analysis and homology search revealed that this protein contained an N-terminal leader region, a GH16 catalytic domain and a C-terminal domain with Ig-like fold. The important role of Ig-like domain has been revealed in many cellulases, but little is known about the biochemical properties of Ig-like domain in bacterial β-1,3-glucanases. To investigate the role of the N- and C-terminal domains in enzymatic function, the *Escherichia coli* (*E. coli*) expression system of PglA and its truncated mutants was constructed in this study. Here, we report the cloning of a new β-1,3-glucanase gene *pglA* from S09, the construction and purification of the truncated enzymes, the comparison of the enzymes in terms of their biochemical characteristics and affinities to insoluble β-1,3-glucans. To the best of our knowledge, this is the first report that describes function activity relationship between catalytic and non-catalytic domains of a bacterial β-1,3-glucanase which containing a C-terminal Ig-like fold.

## Methods

### Strains, media, vectors and chemicals

The strain *Paenibacillus* sp. S09 (CCTCC accession no:M2012196; China Center for Type Culture Collection, Wuhan, China) was originally isolated from the rhizosphere of oat plants. Luria-Berntani (LB) broth supplemented with 0.5% pachyman was used for the production of β-1,3-glucanase by strain S09. *E. coli* competent cell DH5α (Takara, Otsu, Japan) and the plasmid pCR2.1 vector (Invitrogen, Carlsbad, CA, USA) were used for gene cloning. *E. coli* BL21 (DE3) and the pET-29a(+) vector (Novagen, San Diego, CA, USA) were used for gene expression. Recombinant *E. coli* was cultured in LB broth supplemented with kanamycin (50 μg/ml) at 37°C. Recombinant *E. coli* BL21 (DE3) harboring the expression vector was cultivated in 2 × YT medium which containing 1.0% (w/v) yeast extract, 1.6% (w/v) peptone and 0.5% (w/v) NaCl. The His_6_-tagged protein was purified by Ni-sepharose 6 fast flow column (GE healthcare, Sweden). The DNA purification kit, restriction endonucleases, T4 DNA ligase, *pfu* and *LA Taq* DNA polymerase with GC buffer and dNTPs were purchased from Takara. Isopropyl-β-D-thiogalactopyranoside (IPTG) and 5-bromo-4-chloro-3-indolyl β-D-glucuronide (X-Gal) were also purchased from Takara.

Laminarin (from *Laminarium digitatum*), barley β-glucan, zymosan A were purchased from Sigma-Aldrich (Co., St. Louis, USA). Pachyman was purified from commercial fruiting bodies of the basidiomycete *Poria cocos*. Carboxymethyl cellulose-sodium (CMC) and dextran were also purchased from Sigma. Salecan, a novel soluble linear β-1,3-glucan with α-1,3-linkages, were prepared from *Agrobacterium* sp. ZX09, as described by Xiu et al. [16]. All other reagents were of analytical grade.

### Molecular cloning of the β-1,3-glucanase encoding gene

The genomic DNA of strain S09 was extracted as described by Sambrook and Russell [17]. In order to obtain partial of the β-1,3-glucanase gene sequence, degenerate primers (Pdg-F and Pdg-R) was designed based on the two conserved amino acid sequences of GH16 β-1,3-glucanases (Table 1, see Additional file 1: Figure S1). the PCR was performed to amplify a partial β-1,3-glucanase gene as follows: 94°C for 5 min; 30 cycles of 94°C for 30 s, 55°C for 30 s, 72°C for 1 min; one final extension at 72°C for 10 min. The genomic DNA of strain S09 was used as the template. The products of PCR reaction were gel-purified, ligated into pCR2.1 vector, transformed into DH5α and sequenced by Invitrogen. Inverse PCR (I-PCR) and Self-Formed Adaptor PCR (SEFA-PCR) were employed to obtain the full sequence of β-1,3-glucanase gene according to the manufacturer’s instructions [18]. The primers used in I-PCR and SEFA-PCR were listed in Table 1. The primers “gIF1, gIF2, gIR1, gIR2” constructed according the partial β-1,3-glucanase gene were used in I-PCR. Briefly, genomic DNA was digested with *Hind*Ш for 3 h. The digested genomic DNA fragments were gel-purified and subsequently circularized by use of the DNA ligation kit (Takara). I-PCR amplification was conducted with the circulated DNA as template. The primers 5′-sp1, 5′-sp2, and 5′-hemi-sp3 were used to amplify the 5′ flanking sequence, 3′-sp1, 3′-sp2, and 3′-hemi-sp3 were used to amplify the 3′ flanking sequence of the partial β-1,3-glucanase gene. The detailed procedures for SEFA-PCR were shown in Additional file 2. The amplified PCR products were purified, cloned into the pCR2.1 plasmid, and sequenced.

**Table 1:** Primers used for the cloning of β-1,3-glucanase gene

**Primer name**	**Primer sequence (5′ → 3′)**^**a**^
Pdg-F	TAY ACS CGS ACR TAR TC
Pdg-R	GAY TAY GTS CGS GTR TA
gIF1	GTAGCGGTTGACCGGCCATTGG
gIF2	GCTGCTGTTCGCCTTGGAGT
gIR1	CGACCAGCCGTTCTATCTGATCAT
gIR2	GGCGACGATGCAGGTAGACTATGT
5′-sp1	CGTTTCATTGACATTCTGGAAGACGAGCGT
5′-sp2	TGTAGTTGAGTGTGACGCTGCTGTTCGC
5′-hemi-sp3	AATGTCGACCCAGCTNNNNNNNNNCTTGAC
3′-sp1	AGAGATCGACATCATGGAGGCGAAGGG
3′-sp2	TACCTCCCGCAAGGTACGACATTCG
3′-hemi-sp3	ACCAGCCGTTCTATCNNNNNNNNAACCTG

^a^Restriction sites in the primers are italicized. Y = C/T, S = C/G, R = A/G.

### Sequence analysis

Nucleotide sequences were assembled using the DNAMAN software package (Version 5.2.2, Lynnon BioSoft, USA). The signal peptide was predicted using SignalP 4.0 server (http://www.cbs.dtu.dk/services/SignalP) [19]. The alignments of DNA and protein sequences were performed at National Center for Biotechnology Information (NCBI) using the blastn and blastp program (http://www.ncbi.nlm.nih.gov/BLAST/), respectively. The conserved domains and classification of GH family were identified with the website http://www.ncbi.nlm.nih.gov/Structure/cdd/cdd.shtml[20]. Protein secondary structure was predicted using PSIPRED 3.0 [21] (http://bioinf.cs.ucl.ac.uk/psipred). Structures of endo-1,3-β-glucanase from *Cellulosimicrobium cellulans* (PDB ID: 3atgA) and the C-terminal Ig-like domain of the bacteriophage λ tail tube protein (PDB ID: 2l04A) were used as templates for protein structure homology modeling using the automated mode of SWISS-MODEL [22]. Multiple sequence alignments were performed with ClustalX2 [23]. Distance matrix for nucleotides was calculated according to the Kimura’s two-parameter model [24]. Phylogenetic tree, assessed by 1,000 bootstraps, was constructed using neighbor-joining algorithms with MEGA 5.0 software [25].

### Construction of expression vectors

**Figure 1:**
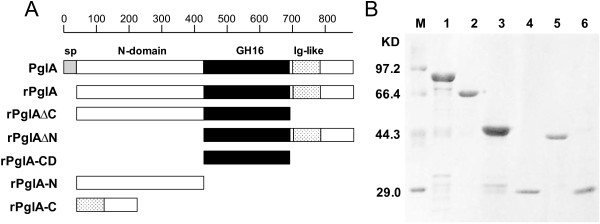
**Schematic overview of PglA and its truncated derivatives. A**, Schematic representation of the module composition of truncated proteins. Abbreviations: sp: singal peptide; ∆X: deletion derivative, with X referring to deleted domain; N: N-terminal leader region (N-domain); CD: GH16 catalytic domain of PglA (GH16); C, C-terminal region containing the Ig-like domain. Every truncated protein has a histidine tag fused to the C-terminus. The scale at the top of the figure indicates amino acid residues. **B**, SDS-PAGE analysis of the purified derivatives of PglA. SDS-PAGE was done using a 12% polyacrylamide gel. Proteins were stained with Coomassie Brilliant Blue R250. Lane M: protein molecular weight marker; Lane 1: rPglA; Lane 2: rPglA∆C; Lane 3: rPglA∆N; Lane 4: rPglA-CD; Lane 5: rPglA-N; Lane 6: rPglA-C.

Five truncated mutants containing one or two modules were designed according to the conserved domain analysis (Figure 1). The corresponding coding regions were amplified using respective pairs of primers by PCR (Table 2). The amplification reactions performed with *pfu* DNA polymerase consisted of 30 cycles of 94°C for 30 s, 60°C for 30 s, 72°C for 1-3 min, followed by a 10 min extension at 72°C. The PCR-amplified fragments were purified and digested with *Kpn*І and *Hind*Ш and then ligated into the expression vector pET-29a(+) with a C-terminal His_6_-tag. After transformation into *E. coli* DH5α, positive transformants were screened and checked by DNA sequencing. The recombinant plasmids were used to transform *E. coli* BL21 (DE3) competent cells for protein expression.

**Table 2:** Primers used for the construction of the expression plasmid

**Construct**	**Primer sequence (5′ → 3′)**^**a**^
PglA	F1 CGGGGTACCGCGACGACCGTGACGTCGAT
	R3 CCCAAGCTTTTACTCAACAACACCTTGTGTTACC
PglA∆C	F1 CGGGGTACCGCGACGACCGTGACGTCGAT
	R2 CCCAAGCTTTTAGCCCGCGCCCTCTTTGTAC
PglA∆N	F2 CGGGGTACCTGGAATCTGTATTGGCAGGATG
	R3 CCCAAGCTTTTACTCAACAACACCTTGTGTTACC
PglA-CD	F2 CGGGGTACCTGGAATCTGTATTGGCAGGATG
	R2 CCCAAGCTTTTAGCCCGCGCCCTCTTTGTAC
PglA-N	F1 CGGGGTACCGCGACGACCGTGACGTCGAT
	R1 CCCAAGCTTTTATCCTGCGATGGCCGGATCG
PglA-C	F3 CGGGGTACCAACGTCGCGGTGACCGGC
	R3 CCCAAGCTTTTACTCAACAACACCTTGTGTTACC

^a^The restriction sites included in the oligonucleotides are underlined.

### Gene expression and protein purification

For recombinant protein production, recombinant *E.coli* BL21 cells harboring the expression vectors were first inoculated into LB medium supplemented with kanamycin (50 μg/ml) and grown overnight at 37°C. Then, the culture was transferred into fresh 2 × YT medium (5:100 dilution) containing 50 μg/ml kanamycin and grown at 37°C to an OD_600_ ≈ 0.8, induced with 0.1 mM IPTG and further cultivated for 6-8 h at 25°C. The culture medium was centrifuged at 12,000 × g for 10 min at 4°C. Cells were washed and suspended in PBS buffer, lysed by sonication on ice and centrifuged at 15,000 × g for 20 min at 4°C to remove the cell debris. The supernatant was applied to Ni-sepharose 6 fast flow column (GE healthcare, Sweden) that had been equilibrated with binding buffer (20 mM sodium phosphate, 500 mM NaCl, pH 7.2). The protein was eluted using a linear gradient of elution buffer (20 mM sodium phosphate, 500 mM NaCl, 15-300 mM imidazole, pH 7.2). Fractions having the β-1,3-glucanase activity were pooled and analyzed by sodium dodecyl sulfate-polyacrylamide gel electrophoresis (SDS-PAGE).

### Enzyme activity assay and protein determination

The β-1,3-glucanase activity assay was carried out by measuring the amount of reducing sugars released from a β-1,3-glucan substrate (laminarin) according to the 3,5-dinitrosalicylic acid (DNS) method [26]. Unless otherwise stated, the reaction mixture was incubated at 55°C for 20 min using laminarin (5 mg/ml) as the substrate in 20 mM sodium acetate buffer (pH 6.0). The amount of reducing sugar was then determined spectrophotometrically at 550 nm. One unit (U) of enzyme activity was defined as the amount of enzyme required to release reducing sugars that equivalent to 1 μmol glucose per min under the test conditions. Hydrolytic activities toward insoluble substrates were performed by gently mixing reaction mixtures during incubation. Then the mixture was centrifuged at 12,000 × g at 4°C for 10 min. The reducing sugars in the supernatant were measured by the DNS method. The protein concentration was measured by the bicinchoninic acid (BCA) method using the BCA protein assay reagent (Thermo Fisher Scientific, MA, USA), Bovine serum albumin (BSA) was used as the standard protein.

### Enzyme characterization

With laminarin as the substrate, the optimal temperature for β-1,3-glucanase activity was determined over the range of 30-80°C in sodium acetate buffer (pH 6.0). Enzyme thermostability was determined by measuring the residual activity after pre-incubation of the enzyme in sodium acetate buffer (pH 6.0) at 55°C without substrate for up to 60 min. The optimal pH for β-1,3-glucanase activity was determined at 55°C in buffers with pH ranging from 2.0 to 10.0. The enzyme stability at different pH was estimated by measuring the residual enzyme activity after incubating the enzyme solution in buffers with pH of 2.0-12.0 at 4°C for 4 h. The buffers (50 mM) used were glycine-HCl (pH 2.0-4.0), sodium acetate (pH 5.0-7.0), Tris–HCl (pH 7.5-8.5) and glycine-NaOH (pH 9.0-12.0).

The substrate specificity was assayed in reactions with 1% laminarin, pachyman, zymosan A, barley β-glucan, CMC, dextran and 0.5% salecan under the optimal conditions for each enzyme. The reaction mixture was often oscillated slightly during incubation for insoluble polysaccharides.

Kinetic study was performed with laminarin as the substrate under the optimal conditions for each enzyme. The initial velocities under laminarin concentrations (0.5-5 mg/ml) were measured, and the values of *K*_m_ and *V*_max_ were obtained from Lineweaver-Burk plots.

### Binding assay

Binding strength of the enzymes to insoluble polysaccharides was estimated by mixing 15-20 μg of the purified proteins with various substrates (10 mg) in sodium acetate buffer (200 μl) at 4°C for 1 h with gentle shaking. Binding mixture without substrate was set as control. The binding ability of enzymes containing catalytic domain to insoluble polysaccharides was determined by measuring the residual enzyme activity in the supernatant of the binding mixture. The amount of the enzyme bound to the polysaccharide was estimated by subtraction of the activity recovered from the original activity [27]. The binding ability of non-catalytic domain to insoluble substrates was also determined [28]. After 1 h of binding reaction, the fraction of protein in the supernatant and the pellet was estimated by 12% SDS-PAGE analysis. BSA was used as the negative control and was handled similarly to the other samples.

### Accession number

The nucleotide sequence for the GH16 β-1,3-glucanase gene *pglA* were deposited in GenBank database under the accession number JX070086.

## Results

### Cloning of the β-1,3-glucanase gene and sequence analysis

**Figure 2:**
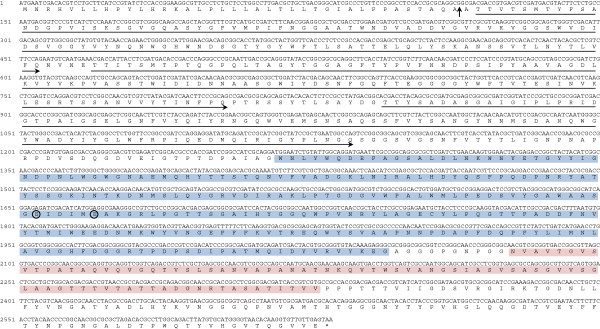
**The nucleotide and amino acid sequences of PglA from *Paenibacillus *sp. S09.** A vertical arrow indicates a cleavage site to produce the mature enzyme. The three N-terminal repeats are indicated by arrow line. The catalytic domain and Ig-like domain are shadowed by blue and red, respectively. The putative catalytic amino acids are circled. The stop codon is indicated by asterisk.

**Figure 3:**
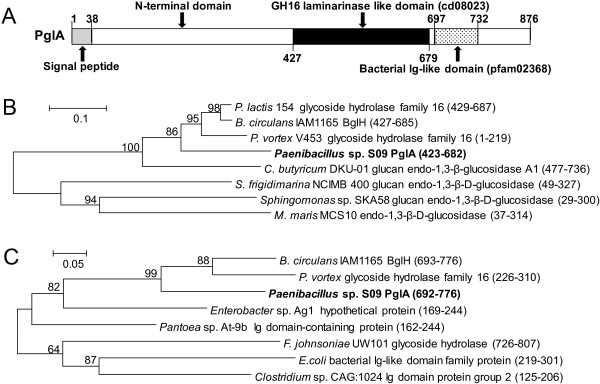
**Sequence analysis of PglA. A**, Schematic representation of PglA with the functional domains. The numbers indicated amino acid positions. The GH16 catalytic domain was indicated in black, signal peptide was indicated in gray, the bacterial Ig-like domain was shown by a stippled box. **B** and **C**, Phylogenetic analysis of the GH16 and Ig-like domains from different proteins, respectively. The amino acid sequence of the GH16 or Ig-like domain was used in BLASTP search and matched sequences were extracted and aligned by ClustalX2. The starting and ending amino acid positions are shown. The unrooted dendrogram was constructed by neighbor-joining method. The percentages of bootstrap values (based on 1000 bootstraps) are shown at the internal nodes. GenBank accession numbers: *P. lactis* 154, EHB65983.1; *B. circulans* BglH, AAC60453.1; *P. vortex*, EFU40835.1; *C. butyricum* DKU-01, EMU55784.1; *S. frigidimarina* NCIMB 400, ABI71172.1; *Sphingomonas* sp. SKA58, EAT10234.1, *M. maris* MCS10, ABI64540.1; *Enterobacter* sp. Ag1, EJF31699.1; *Pantoea* sp. At-9b, ADU69429.1; *F. johnsoniae* UW101, ABQ04240.2; *E.coli*, EMX44285.1; *Clostridium* sp. CAG:1024, CCX42470.1.

A β-1,3-glucanase-encoding gene designated *PglA* was identified from a 5053-bp fragment which was obtained by using degenerate PCR, I-PCR and SEFA-PCR. The deduced amino acid sequence of PglA (GenBank No.: AFO67889.1) comprises 876 amino acids with a predicted N-terminal signal peptide (residues 1-38) (Figure 2). The mature protein consists of 838 amino acid residues with a calculated pI of 4.5 and molecular mass of 90.4 kDa, respectively. The deduced amino acid sequence of PglA exhibited the highest identity (86%) with the hypothetical protein from *Paenibacillus daejeonensi*s (NCBI Reference Sequence: WP_020617344.1), 77% identity with the glycoside hydrolase family 16 protein from *Paenibacillus lactis* 154 (GenBank No.: EHB65983.1), and 76% identity with the reported β-1,3-glucanase BglH from *Bacillus circulans* IAM1165 (GenBank No.: AAC60453.1). PglA showed only 45% identity with the well-characterized endo-β-1,3-glucanase LamA from *Paenibacillus* sp. CCRC 17245 (GenBank No.: ABJ15796.1). Combing the results of conserved domain search in the Conserved Domain Database (CDD) of NCBI, secondary structure prediction and tertiary structure homology modeling by SWISS-MODEL (See Additional file 3: Figure S2), PglA contains a GH16 laminarinase-like domain and a bacterial Ig-like domain (Figures 2 and 3A). The domain organization of PglA is not similar to LamA, which include a leader peptide, a threefold repeat of S-layer homologous module, a GH16 catalytic module, four repeats of CBM_4_9 and an analogue of coagulation factor Fa5/8C from N to C terminus [13]. PglA could be considered as a novel β-1,3-glucanase of *Paenibacillus* species. Phylogenetic tree showed that the catalytic region of PglA forms a distinct clade with BglH from *B. circulans* and putative glycoside hydrolases from *Paenibacillus* species (Figure 3B). Phylogenetic analysis based on the Ig-like domain sequence alignment indicated the relationships between PglA and other Ig-like domain containing proteins (Figure 3C).

### Expression of full-length and truncated *PglA* in *E.coli* and protein purification

Using basic molecular cloning techniques, the gene fragment of *PglA* without the signal peptide sequence and five truncated mutants were cloned into pET-29a(+) vector and successfully expressed in *E.coli* BL21 (DE3). Figure 1A represents a schematic overview of the recombinantly produced constructs. The His_6_-tagged enzymes were purified by affinity chromatography on a Ni-sepharose 6 fast flow column. Molecular masses of rPglA and its truncated mutants rPglA∆C, rPglA∆N, rPglA-CD, rPglA-N and rPglA-C were estimated to be 91 kDa, 70 kDa, 49 kDa, 29 kDa, 42 kDa and 21 kDa, respectively. SDS-PAGE analysis indicated protein bands with molecular masses corresponding well to the theoretical values. All the enzymes were purified to a homogeneous state on SDS-PAGE (Figure 1B). The steps involved in a typical purification process are summarized in Table 3.

**Table 3:** Purification of rPglA and its truncated derivatives

**Purification step**	**Total activity (U)**	**Total protein (mg)**	**Specific activity (U/mg)**	**Yield (%)**	**Purification (fold)**
**rPglA**					
Crude extract	2.56	9.80	0.26	100	1
Ni2 + −affinity column	1.43	0.72	1.99	58.1	7.62
**rPglA∆C**					
Crude extract	4.01	12.4	0.32	100	1
Ni2 + −affinity column	2.21	1.05	2.14	55.1	6.63
**rPglA∆N**					
Crude extract	2.85	9.71	0.29	100	1
Ni2 + −affinity column	1.53	0.75	2.04	53.7	6.96
**rPglA-CD**					
Crude extract	2.51	8.81	0.29	100	1
Ni2 + −affinity column	1.05	0.60	1.76	42.1	6.17

### Effects of temperature and pH on the enzymatic activity and stability

The general properties including the optimal pH and temperature of PglA and the truncated derivatives were investigated with laminarin as the substrate. Deletion of the N-or C-terminal domain did not abolish the hydrolytic function of β-1,3-glucanase, thus, enzymatic properties of rPglA, rPglA∆C, rPglA∆N and rPglA-CD were characterized respectively.

**Figure 4:**
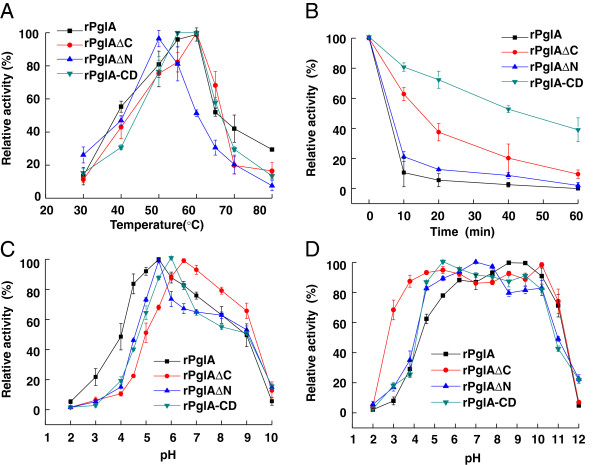
**Enzymatic characteristics of purified rPglA and the truncated enzymes. A**, Influence of temperature on β-1,3-glucanase activity. Reactions were carried out at pH 6.0 and temperatures ranging from 35 to 80°C for 20 min. Values were expressed as percentages of maximal activity. **B**, Thermal stability of rPglA and the truncated derivatives. The enzymes were incubated at 55°C for up to 60 min and the residue enzyme activities were determined under optimal conditions for 20 min. **C**, Influence of pH on β-1,3-glucanase activity. Reactions were carried out at optimal temperatures for each enzyme for 20 min in buffers of varying pH (2.0-10.0). **D**, pH stability of rPglA and the truncated derivatives. Purified enzymes were first incubated in buffers of varying pH (2.0-12.0) at 4°C for 4 h, and activity was measured under optimal conditions for 20 min. The buffers (50 mM) used were: glycine-HCl (pH 2.0-4.0), sodium acetate (pH 5.0-7.0), Tris–HCl (pH 7.5-8.5) and glycine-NaOH (pH 9.0-12.0). The measurements were repeated in triplicate.

Effect of domains on thermoactivity and thermostability of rPglA was investigated. Side by side comparison of the temperature activity profiles of rPglA and the truncated mutants (rPglA∆C, rPglA∆N and rPglA-CD) showed that, rPglA∆N, the PglA variant containing the catalytic domain and the C-terminal Ig-like domain, obviously is the least thermoactive enzyme. It had its highest activity at 50°C, while, rPglA, rPglA∆C and rPglA-CD have a maximum activity at 60°C (Figure 4A). This phenomenon indicated that the deletion of the C-terminus increased the optimal temperature. As a dramatic loss of enzymatic stability occurred in the full length enzyme (rPglA) at temperatures higher than 60°C, the thermal stability was assayed at 55°C for up to 60 min. In conjunction with the temperature optima, also the thermal stability of the truncated derivatives of PglA turned out to be module related. As observed by the thermal stability assay, the absence of C-terminal region is of great relevance for higher temperature stability of the proteins. This can be deduced from the unexpected higher stability of rPglA-CD and rPglA∆C, and lower stability of rPglA and rPglA∆N (Figure 4B). These results indicated that truncation of C-terminal domain improved the thermostability of PglA.

Deletion of the N-or C-terminal regions had no drastic effect on the optimal pH. For all PglA derived constructs, an apparent pH optimum between pH 5.5 and 6.5 was observed (Figure 4C). rPglA and the truncated enzymes showed broad pH stability. All enzymes retained more than 80% of their original enzymatic activity after 4 h incubation in buffers with pH values between pH 5.0 and 10.0 (Figure 4D). It should be noted that the C-terminal domain deleted mutant rPglA∆C retained higher activity in the acidic range of the pH spectrum (pH 2.0-4.5) than the other deletion derivatives.

### Substrate specificity and kinetic analysis

The purified rPglA and truncated enzymes (PglA∆C, PglA∆N and PglA-CD) shared similar substrate specificity. They were highly active toward soluble β-1,3-glucan (laminarin) and insoluble β-1,3-glucan (pachyman and zymosan A). Low activity was detected on soluble linear glucan, barley β-glucan which contains mixed β-1,3-and β-1,4- linkages, and salecan which contains mixed β-1,3-and α-1,3-linkages, little activity on CMC, dextran and xylan (Table 4). It was not surprising as β-1,3-glucan specificity is the general rule for laminarinases. Side by side comparison of the specific activity of rPglA and the truncated mutants (rPglA∆C, rPglA∆N and rPglA-CD) showed that, both the N-and C-terminal domains could enhance the hydrolyzing activity of the catalytic module toward insoluble substrates. It was worth noting that rPglA∆C and rPglA-CD show slightly higher activity toward laminarin than rPglA and rPglA∆N. It should be related to the improved thermostability of rPglA∆C and rPglA-CD as the deletion of the C-terminal region.

**Table 4:** Substrate specificity of rPglA and its truncated derivatives

**Substrate**^**a**^	**Solubility**	**Main linkage**		** Specific activity (U/mg)**^**b **^**of:**		
			**rPglA**	**rPglA∆C**	**rPglA∆N**	**rPglA-CD**
Laminarin	Soluble	β-1,3, β-1,6 (Glc)	2.016 ± 0.213	2.506 ± 0.131	2.000 ± 0.099	2.525 ± 0.141
Pachyman	Insoluble	β-1,3 (Glc)	1.695 ± 0.165	2.396 ± 0.339	1.883 ± 0.045	0.898 ± 0.078
Zymosan A	Insoluble	β-1,3, β-1,6 (Glc)	1.364 ± 0.131	1.518 ± 0.036	0.835 ± 0.022	0.567 ± 0.035
Barley β-glucan	Soluble	β-1,3, β-1,4 (Glc)	0.086 ± 0.011	0.074 ± 0.001	0.076 ± 0.021	0.081 ± 0.015
Salecan	Soluble	β-1,3, α-1,3 (Glc)	0.049 ± 0.010	0.039 ± 0.005	0.037 ± 0.009	0.032 ± 0.007
CMC	Soluble	β-1,4 (Glc)	0	0	0	0
Dextran	Soluble	α-1,6 (Glc)	0	0	0	0
xylan	Insoluble	β-1,4 (xyl)	0	0	0	0

^a^All substrates were used at a final concentration of 10 mg/ml, except salecan (5 mg/ml).

^b^Activity ± standard error of triplicate samples.

The kinetic parameters with laminarin as substrate were determined from substrate concentration dependence of velocity. Based on double reciprocal plots, the values for the Michaelis constant (*K*_m_), turnover number (*k*_cat_) and catalytic efficiency (*k*_cat_/*K*_m_) are calculated and presented in Table 5. *K*_m_ values of rPglA, rPglA∆C, rPglA∆N and rPglA-CD were calculated to be 0.94, 1.01, 1.36 and 2.64 mg/ml, respectively, suggesting that deleting N-and C-terminal domains caused significantly increased *K*_m_ values. The *k*_cat_/*K*_m_ values of rPglA∆C, rPglA∆N and rPglA-CD were about 70%, 78% and 35% of that of rPglA respectively. These kinetic parameters indicate that the N-and C-terminal domains increase the affinity of PglA for the substrate (laminarin) and thus enhance the catalytic efficiency of catalytic module.

**Table 5:** **Kinetic parameters**^**a **^**of rPglA and its truncated derivatives toward laminarin**

**Enzyme**	***K***_**m **_**(mg/ml)**	***k***_**cat **_**(s**^**-1**^**)**	***k***_**cat**_**/*K***_**m **_**(s**^**-1**^ **· mg**^**-1**^ **· ml)**
rPglA	0.94 ± 0.07	3.56 ± 0.18	3.79 ± 0.31
rPglA∆C	1.01 ± 0.05	2.68 ± 0.07	2.66 ± 0.09
rPglA∆N	1.36 ± 0.11	4.01 ± 0.10	2.95 ± 0.07
rPglA-CD	2.64 ± 0.15	3.54 ± 0.21	1.34 ± 0.07

^a^Data are shown as mean ± SD (n = 3).

### Binding assay

**Figure 5:**
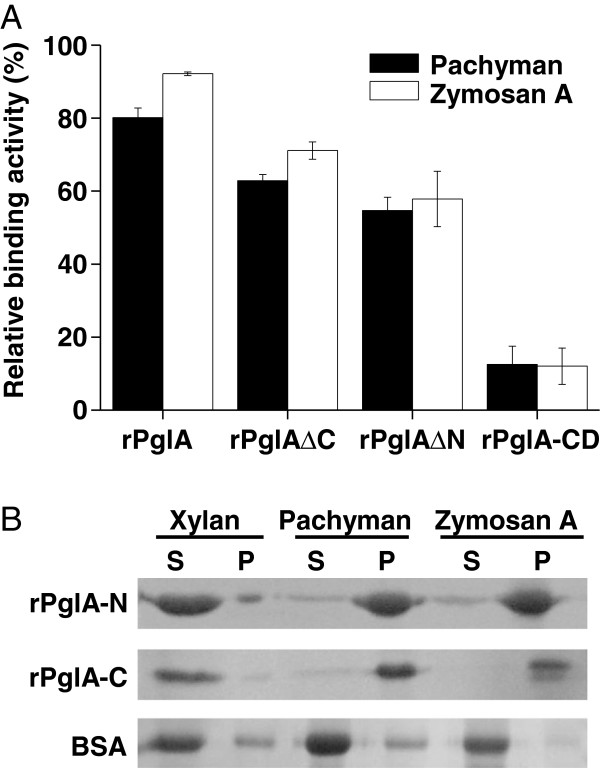
**Binding assay of the truncated enzymes toward insoluble polysaccharides.** The purified protein (15-20 μg) and the indicated substrate (10 mg) were thoroughly mixed at 4°C for 1 h. **A**, Binding assay of the four active enzymes (PglA, rPglA∆C, rPglA∆N, rPglA-CD). The enzymatic activity against laminarin remaining in the supernatant was determined and compared to the original activity of each enzyme preparation. The binding activity was calculated by subtraction of the activity recovered from the original activity which was considered as 100%. The values represent the average of the results from triplicate experiments. **B**, Binding of rPglA-N and rPglA-C to insoluble carbohydrates. The amount of protein remaining in the supernatant (S) and co-precipitating with the substrate (P) were examined by SDS-PAGE.

Binding experiments with rPglA and the truncated derivatives and BSA as the tested protein were performed. Two approaches were taken to explore the polysaccharide-binding function of the N-terminal and C-terminal regions. First, as for rPglA and the truncated enzymes containing the catalytic domain (rPglA∆C, rPglA∆N, rPglA-CD), residual enzymatic activity in the supernatant of binding mixtures was measured. As shown in Figure 5A, the catalytic domain of PglA (rPglA-CD) had the minimum binding ability compared to the other three enzymes. Both the N-and C-terminal regions of PglA had significantly carbohydrate-binding activities. These results were confirmed by the insoluble carbohydrate-binding analysis of rPglA-N and rPglA-C. The N-and C-terminal domain bound strongly to insoluble β-1,3-glucan (pachyman and zymosan A), as a large fraction of the protein was retained in the pellet fraction, but not to xylan (Figure 5B). Presence of insoluble polysaccharide did not affect the amount of BSA in supernatant, excluding the nonspecific binding of insoluble polysaccharide to protein.

## Discussion

Enzymes that hydrolyze insoluble complex polysaccharide structures are usually multi-modular enzymes and composed of distinct catalytic domain and non-catalytic carbohydrate binding modules (CBMs) that play a pivotal role in the action of these enzymes against recalcitrant substrates [29]. The presently known CBMs are classified into 67 families in the latest update of the CAZy database (http://www.cazy.org/fam/acc_CBM.html). Among the 67 CBMs families classified in CAZy, one or more of CBMs of 4, 6, 9, 13, 54, 56 families have been found in bacterial β-1,3-glucanases [13,27,29-31]. In the work reported here, a novel β-1,3-glucanase gene *PglA* was cloned from *Paenibacillus* sp.S09. PglA contains three functional regions: an N-terminal domain, a GH16 catalytic domain, and a C-terminal domain with an Ig-like structure. Even high sequence similarity was found between PglA and the β-1,3-glucanase BglH from *Bacillus circulans* IAM1165, PglA possessed a novel Ig-like domain which was not observed in BglH by conserved domain search in NCBI. Previous reports demonstrated carbohydrate-binding properties of the N-and C-terminal regions of BglH [27,32], while, the effects of the non-catalytic domain on enzymatic properties of BglH were not described. Furthermore, the enzymatic properties of Ig-like domain, which is generally considered being involved in carbohydrate binding and commonly found in bacterial proteins [33], have not been illustrated in bacterial β-1,3-glucanases. We were interested to investigate the functional structural elements of PglA.

It is generally believed that each functional module in a polypeptide can function normally even after separation from an adjacent module [34]. The full-length PglA and five truncated derivatives containing one or two modules were constructed, recombinantly produced, purified and characterized. The function of each module referring to carbohydrate-binding and hydrolyzing properties was identified respectively. Specific activities of PglA and truncated derivatives on various substrates were measured. The strict and selective specificity of PglA suggested that it was a strict β-1,3-glucanase belonging to EC 3.2.1.39 family of glycoside hydrolases. The truncated mutant PglA-CD, which contained only the catalytic domain, showed reduced hydrolytic activity on insoluble substrates, while, its activity remained unchanged on soluble substrates (Table 4). It could be suggested that the N- or C-terminal domain did not play a direct or critical role in the structural stability of the catalytic domain. The results of binding assay demonstrated that both the N-and C-terminal domains of PglA had significant binding activities toward insoluble linear β-1,3-glucan (pachyman) and branched-chain β-1,3-glucan (zymosan A) respectively (Figure 5), suggesting their module functions in the enzymatic activity of PglA. It was in accordance with the β-1,3-glucanase (BglH) from *Bacillus circulans* IAM1165 [11]. Amino acid sequence alignment showed no significant similarity between the N-and C-terminal domains. Thus, both of them could be classified into CBM families respectively. Either N-or C-terminal CBM could exert the strong binding toward insoluble β-1,3-glucan (pachyman and zymosan A), which probably account for the increase of the specific activities of rPglA∆C or rPglA∆N against these insoluble substrates, presumably by assisting the localization of catalytic domains to insoluble polysaccharides.

The full-length PglA and truncated glucanases (PglA∆C, PglA∆N and PglA-CD) exhibited similar enzymatic properties. The pH activity and stability profiles of rPglA and the truncated enzymes were nearly indistinguishable, except that rPglA∆C retained higher activity in acidic range of the pH spectrum (pH 2.0-4.5) than the other deletion derivatives. The results of thermal activity and stability assay indicated the relatively low influence of the N-terminal CBM on enzymatic properties of PglA. However, deletion of the C-terminus in PglA, consisting of an Ig-like fold and a basic terminal domain, significantly increased the thermal tolerance of the enzyme, which was first observed with bacterial β-1,3-glucanase. These observations imply the negative impact of the C-terminal CBM on enzymatic acidic and thermal stabilities of catalytic domain. A similar negative effect has been identified for endo-β-1,4-glucanase from *Bacillus subtilis* JA18 in that truncation of the cellulose binding domain improved thermal stability of the enzyme [35]. Carbohydrate binding is not the sole function of the non-catalytic modules [36]. The impact of CBMs on enzymatic thermal stability of a multidomain xylanase has been discovered by Winterhalter et al. in 1995 [37]. Increasing literature showed controversial data on CBMs affecting glycoside hydrolase’s thermostability. The comparison of catalytic module alone with catalytic module fused to CBM showed either improvement of thermostability [36-38], or on the contrary appeared to have a negative impact [35,39]. In this report, the negative impact of C-terminal CBM containing an Ig-like structure on thermostability as well as acidic stability of β-1,3-glucanase was revealed.

Proteins possessing the Ig-like fold are commonly found in bacteria, and are most often involved in cell-cell adhesion, or extracellular glycohydrolysis [40]. Recent years, there is increasing structural information on the existence of these domains in glycoside hydrolase family proteins. Ig-like domain is generally considered being involved in carbohydrate binding and assist in hydrolysis reactions for insoluble substrates [33]. The influence of Ig-like domain on enzymatic stability and activity of GH family proteins has been mainly illustrated in cellulose hydrolases. Liu et al. revealed that deletion of the C-terminal Ig-like domains in GH5 family cellulose resulted in a significant loss of enzymatic activity toward soluble and insoluble cellulose substrates [41]. Many members of family 9 of glycoside hydrolases have an N-terminal Ig-like domain which is responsible for the hydrolytic activity and stability of the catalytic domain [33,42]. Tight structural interaction between the Ig-like and GH9 domains was evidenced by analyzing the crystal structure of the Ig-GH9 module pair [42]. It can be inferred that the C-terminal Ig-like structure also physically associate with the active site of the catalytic domain of PglA and hampered the thermal activity and stability of the enzyme. In contrast, the negligible impact of the N-terminal peptide on enzymatic stability and activity indicated it is very likely to fold as an independent structural module and show little interactions with the catalytic module of PglA.

## Conclusions

In the present study, a new β-1,3-glucanase gene *PglA* was cloned from *Paenibacillus* sp. S09. Truncation forms of PglA were successfully expressed in *E.coli*. Both the N-and C-terminal non-catalytic domains exerted carbohydrate-binding functions, while the C-terminal region containing an Ig-like fold had negative impact on thermostability of catalytic domain. There is increasing documentation that the CBMs of bacterial glycoside hydrolases containing Ig-like structure. As the first report describing the characterization of a GH16 β-1,3-glucanase with an C-terminus Ig-like region, the present study improved our understanding on the module relationships between the enzyme’s catalytic and non-catalytic domains and could provide information that may suggest an approach to rationally modify the characteristics of an enzyme for industrial application.

## Abbreviations

GH16: Glycoside hydrolase family 16; I-PCR: Inverse PCR; SEFA-PCR: Self-formed adaptor PCR; CMC: Carboxymethyl cellulose; DNS: Dinitrosalycilic acid; CBM: Carbohydrate-binding module; PglA: Full-length *Paenibacillus* sp. S09 β-1,3-glucanase; PglA∆C: C-terminus truncated PglA; PglA∆N: N-terminus truncated PglA; PglA-CD: Catalytic domain of PglA; PglA-N: N-terminal domain of PglA; PglA-C: C-terminal domain of PglA.

## Competing interests

All of authors declare no competing interests.

## Authors’ contributions

RC performed the cloning, expression and purification experiments, analyzed the data and was involved in drafting the manuscript. JPC and XHY carried out the enzyme activity assays. SMW participated in the sequence analysis and truncation design. YW assisted with sequence analysis and enzyme characterization. JFZ contributed to study conception and design, analysis and interpretation of data, as well as drafting and revision of the manuscript, and acquisition of funding. All authors read and approved the final manuscript.

## Supplementary Material

Additional file 1: Figure S1 Conserved domain sequence alignment of GH16 endo-β-1,3-glucanases. The organisms and accession numbers are as follows: *Nocardiopsis* sp*.*F96 (BAE54302.1), *Cellulosimicrobium cellulans* (AAC44371.1), *Paenibacillus mucilaginosus* KNP414 (YP_004643413.1), *Bacillus clausii* KSM-K16 (YP_174203.1), *Bacillus circulans* IAM1165 (BAA04469.1), *Paenibacillus* sp.CCRC 17245 (ABJ15796.1), *Phaeodactylum tricornutum* CCAP 1055/1 (XP_002181321.1). The most conserved regions were used for degenerated primers. Identical amino acids are indicated by solid black and gray. The GH16 active site and two glutamate residues are indicated by frame and asterisks respectively. Click here for file

Additional file 2 **The detailed procedures of SEFA-PCR method for amplification of β-1,3-glucanase gene.** Click here for file

Additional file 3: Figure S2 Protein structure prediction of PglA. (A) Tertiary structure homology modeling. The PDB IDs of the templates used for modeling were shown in parentheses; (B) Secondary structure prediction by PSIPRED 3.0 with α-helixes shown in orange and β-sheets in blue. Click here for file
