# Supplementary figures and images for: Recombinant production and characterization of full-length and truncated β-1,3-glucanase PglA from Paenibacillus sp. S09
Source: BMC Biotechnol. 2013 Nov 28;13:105. doi: 10.1186/1472-6750-13-105 (PMC4219603; doi:10.1186/1472-6750-13-105)

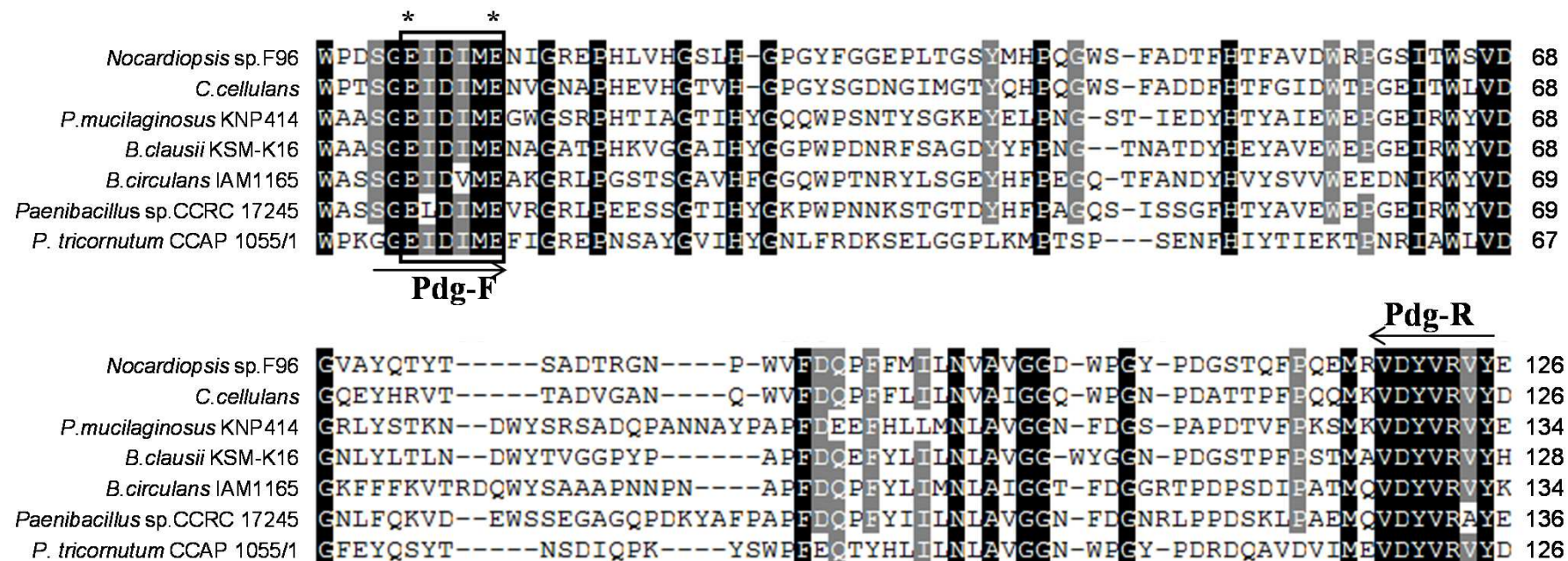

Figure S1

Supplement: Additional file 1: Figure S1 — Conserved domain sequence alignment of GH16 endo-β-1,3-glucanases. The organisms and accession numbers are as follows: Nocardiopsis sp.F96 (BAE54302.1), Cellulosimicrobium cellulans (AAC44371.1), Paenibacillus mucilaginosus KNP414 (YP_004643413.1), Bacillus clausii KSM-K16 (YP_174203.1), Bacillus circulans IAM1165 (BAA04469.1), Paenibacillus sp.CCRC 17245 (ABJ15796.1), Phaeodactylum tricornutum CCAP 1055/1 (XP_002181321.1). The most conserved regions were used for degenerated primers. Identical amino acids are indicated by solid black and gray. The GH16 active site and two glutamate residues are indicated by frame and asterisks respectively. [file 1472-6750-13-105-S1.pdf]

**A**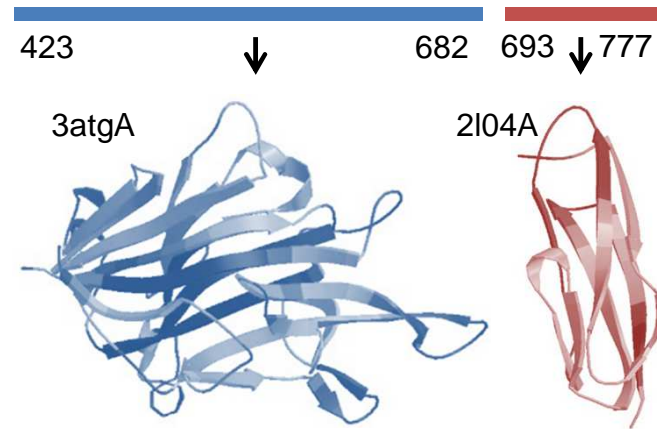**B**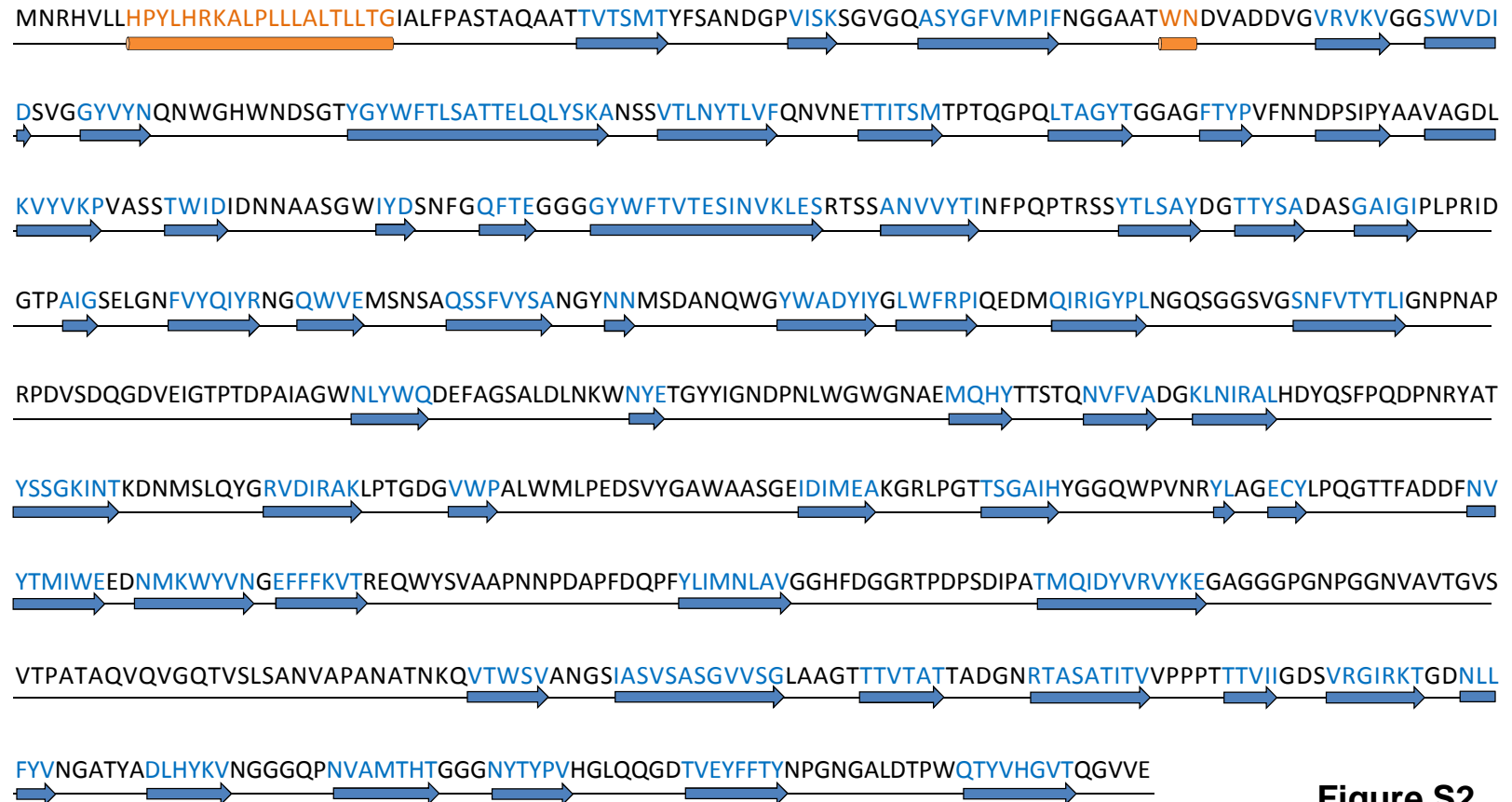**Figure S2**

Supplement: Additional file 3: Figure S2 — Protein structure prediction of PglA. (A) Tertiary structure homology modeling. The PDB IDs of the templates used for modeling were shown in parentheses; (B) Secondary structure prediction by PSIPRED 3.0 with α-helixes shown in orange and β-sheets in blue. [file 1472-6750-13-105-S3.pdf]
