# Supplementary material for: Recombinant production and characterization of full-length and truncated β-1,3-glucanase PglA from Paenibacillus sp. S09
Source: BMC Biotechnol. 2013 Nov 28;13:105. doi: 10.1186/1472-6750-13-105 (PMC4219603; doi:10.1186/1472-6750-13-105)
Supplement: Additional file 2 — The detailed procedures of SEFA-PCR method for amplification of β-1,3-glucanase gene. [file 1472-6750-13-105-S2.pdf]

Additional file 3:

The detailed procedures of SEFA-PCR were as follows.

The PCR mixture included 10 μl of 2 × GC buffer I, 2 μl of 2.5 mM dNTPs, 1 U of LA *Taq*, and about 20 ng of template genomic DNA, with deionized water added to 20 μl. First, a single PCR cycle was carried out using hemi-sp3 as a primer, genomic DNA from S09 as the template, and the following conditions: (1) denaturing at 94°C for 90 s; (2) annealing at 38°C for 3 min, then ramping to 72°C, increasing by 0.2°C per second; and (3) extension at 72°C for 5 min. Second, sp1 primer was added to the reaction mixture, and 25 cycles of PCR were carried out with the following conditions: (1) denaturing at 94 °C for 30 s and (2) annealing and extension at 70°C for 5 min. Third, 10 cycles of thermal asymmetric PCR were carried out with the following conditions: (1) 2 cycles of denaturing at 94°C for 30 s, annealing and extension at 55°C for 5 min, and (2) one cycle of denaturing at 94°C for 30 s, annealing at 55°C for 30 s, and extension at 72 °C for 5 min. Finally, nested PCR was run with the primer sp2, and the above PCR products as a template. Normal PCR were carried out with the following conditions: 94°C for 3 min; 30 cycles of 94°C for 30 s, 60°C for 30 s, 72°C for 5 min; one final extension at 72°C for 10 min.
